# Recent advances in steroidal glycoalkaloid biosynthesis in the genus *Solanum*

**DOI:** 10.5511/plantbiotechnology.23.0717b

**Published:** 2023-09-25

**Authors:** Ryota Akiyama, Naoyuki Umemoto, Masaharu Mizutani

**Affiliations:** 1 Graduate School of Agricultural Science, Kobe University, Hyogo 657-8501, Japan; 2 RIKEN Center for Sustainable Resource Science, Kanagawa 230-0045, Japan

**Keywords:** cytochrome P450, genome editing, steroidal glycoalkaloid, 2-oxoglutarate-dependent dioxygenase

## Abstract

Steroidal glycoalkaloids (SGAs) are specialized metabolites found in members of *Solanum* species, and are also known as toxic substances in *Solanum* food crops such as tomato (*Solanum lycopersicum*), potato (*Solanum tuberosum*), and eggplant (*Solanum melongena*). SGA biosynthesis can be divided into two main parts: formation of steroidal aglycones, which are derived from cholesterol, and glycosylation at the C-3 hydroxy group. This review focuses on recent studies that shed light on the complete process of the aglycone formation in SGA biosynthesis and structural diversification of SGAs by duplicated dioxygenases, as well as the development of non-toxic potatoes through genome editing using these findings.

## Introduction

**Figure 1:**
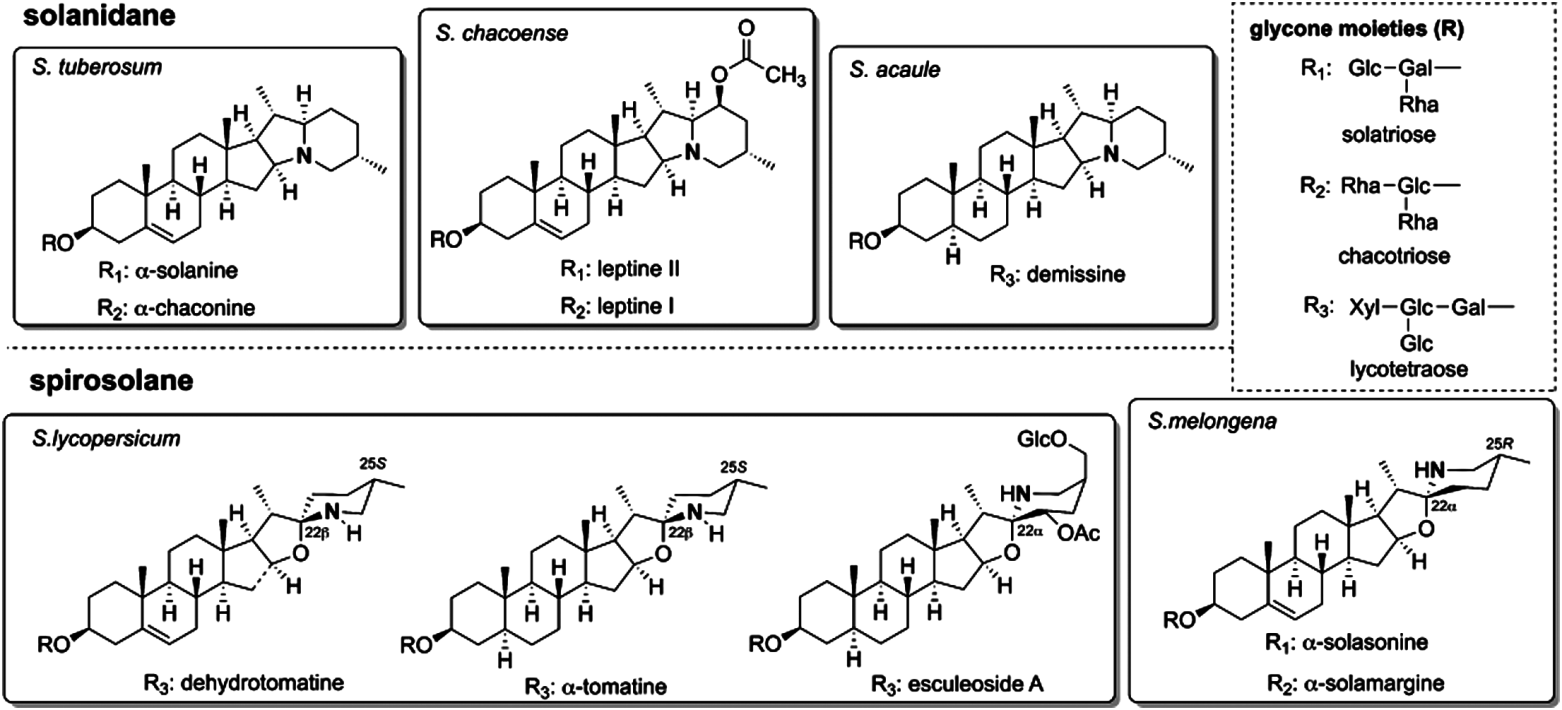
Figure 1. Steroidal glycoalkaloids in *Solanum* spp.

Steroidal glycoalkaloids (SGAs) are typically found in members of *Solanum* species, and are known as toxic substances in *Solanum* food crops (Harrison 1990; Helmut 1998; Petersen et al. 1993) such as tomato (*Solanum lycopersicum*), potato (*Solanum tuberosum*), and eggplant (*Solanum melongena*) (Figure 1). Because of their toxic effects on fungi, bacteria, insects, and animals, SGAs are considered to play defensive roles against a wide range of pathogens and predators (Friedman 2002, 2006). Potatoes are produced worldwide and are the fourth most important crop produced globally, and however, potatoes are known to contain toxic SGAs, such as α-solanine and α-chaconine. SGAs are primarily found in sprouts and green potatoes (particularly near the skin), and their accumulation increases with improper potato management, such as exposure to light. While small amounts of SGAs cause only an unpleasant taste, it can cause food poisoning when ingested in large quantities. Tomatoes contain α-tomatine and dehydrotomatine as major SGAs in green tissues such as leaves and immature fruits (Friedman 2002). However, during tomato fruits ripening, α-tomatine accumulated in immature fruits is metabolized and converted to the non-toxic and non-bitter SGA esculeoside A (Iijima et al. 2009). Eggplants mainly produces α-solasonine and α-solamargine (Sánchez-Mata et al. 2010). In addition, diverse SGAs, such as demissine (*S. acaule*) and leptine I and II (*S. chacoense*) have been reported in wild species of *Solanum* crops (Iijima et al. 2013; Kozukue et al. 2008; Shakya and Navarre 2008) (Figure 1).

SGAs consist of two structural components, C_27_ steroidal alkaloids and oligosaccharides bound to the C-3 hydroxy group, and the enormous structural diversity of SGAs in *Solanum* species is generated by various combinations of steroidal aglycones and sugar moieties. SGAs are biosynthesized from cholesterol, via oxidation at C-16, C-22, C-26 and transamination at C-26, cyclization of EF-ring, and glycosylation at the C-3 hydroxy group. SGA biosynthesis can be divided into two main parts: aglycone formation and glycosylation. Several glycosyltransferases involved in the glycosylation steps of SGA biosynthesis have been identified in potato and tomato (Zhao et al. 2021). This review focuses on recent research that shed light on the complete process of the aglycone formation in SGA biosynthesis and introduces the development of non-toxic potatoes using this knowledge by genome editing.

## Cholesterol biosynthesis genes

**Figure 2:**
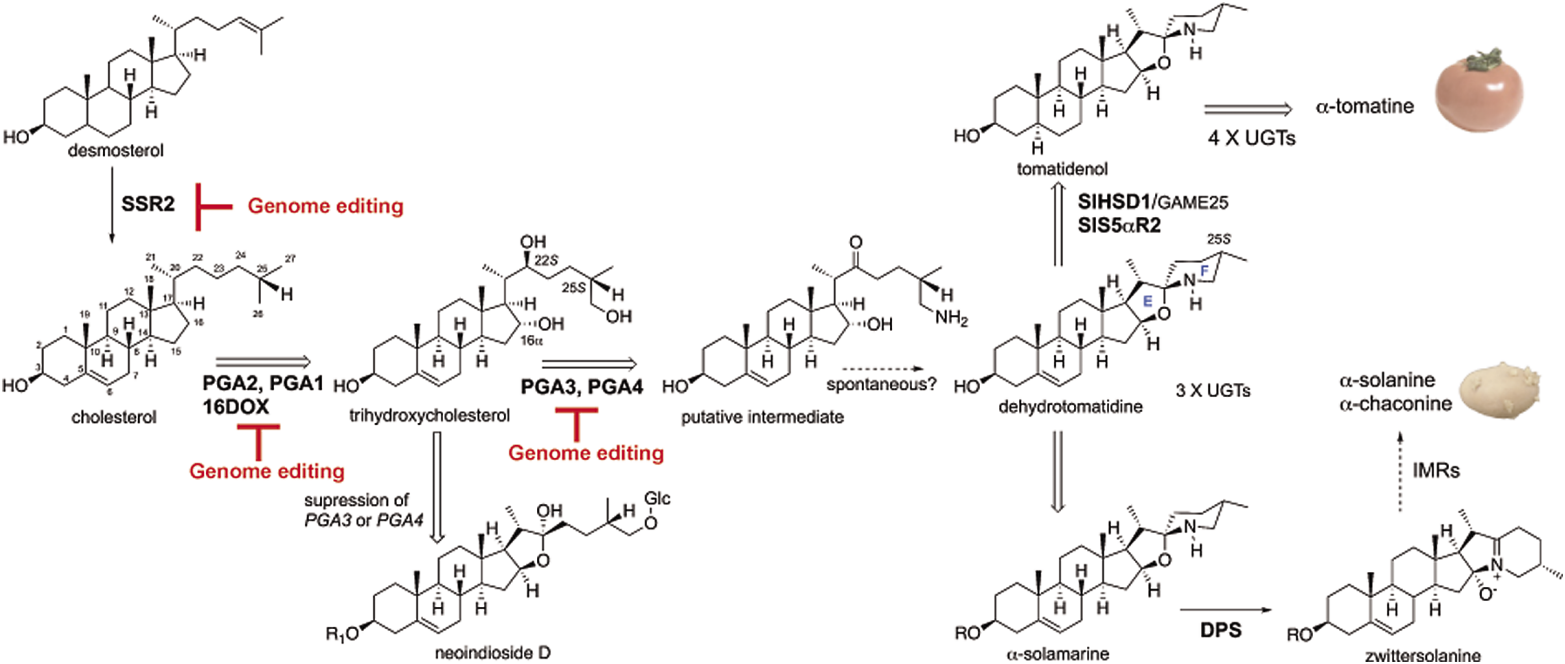
Figure 2. The biosynthetic pathways of steroidal glycoalkaloids in potatoes and tomatoes. The solid thick black arrows indicate the single reaction step, and the names of the enzymes presented in this review are in bold. The double-lined arrows indicate the multiple reaction steps, and the names of the enzymes presented in this review are in bold. The open thick arrow indicates the accumulation of the furostanol saponin neoindioside D by suppression of *PGA3* or *PGA4*. The dotted arrows indicate the putative reaction steps. The red lines indicate possible targets for genome editing that blocks SGA biosynthesis in potatoes.

Tracer experiments suggested that the precursor of SGA is cholesterol. Generally, the main sterols in most plants are C-24 alkyl phytosterols (such as C_29_ sitosterol, stigmasterol and C_28_ campesterol), and the content of C_27_ cholesterol is very low. On the other hand, it is known that the amount of cholesterol in Solanaceae plants including potatoes and tomatoes is high. The difference in chemical structure between C-24 alkyl phytosterols and cholesterol is the presence or absence of an alkyl group at position C-24, which is generated by a methyltransfer reaction and a C-24 reduction reaction. In *Arabidopsis thaliana*, DWF1 has been identified as an enzyme that catalyzes the C-24 reduction reaction in phytosterol biosynthesis (Choe et al. 1999). Analysis of potatoes and tomatoes revealed the existence of two genes that are homologous to *A. thaliana DWF1*, named *SSR1* and *SSR2*, respectively (Sawai et al. 2014). Enzyme activity analysis using yeast showed that SSR1 mainly catalyzes the C-24(28) reduction reaction involved in C-24 alkyl phytosterol biosynthesis, while SSR2 mainly catalyzes the C-24(25) reduction reaction involved in cholesterol biosynthesis (Figure 2). Furthermore, when the *SSR2* expression was suppressed in potatoes, the accumulation of cholesterol and its derivative SGA was greatly reduced. These findings indicate that SSR2 is a key enzyme involved in the branch point of C-24 alkyl phytosterol and cholesterol biosynthesis in potatoes and tomatoes (Sawai et al. 2014). In other words, in potatoes and tomatoes, it is considered that the duplication of a plant’s universal 24(28) reductase gene resulted in the specialization of SSR2 for cholesterol biosynthesis due to changes in enzyme function. In addition, while most of the sterol biosynthesis genes in *A. thaliana* exist as a single copy, many sterol biosynthesis genes in tomatoes and potatoes exist as two or more copies (Sonawane et al. 2016), suggesting that the duplication of sterol biosynthesis genes in Solanaceae plants supports the high cholesterol production.

## Cluster of SGA biosynthesis genes

Through comparative co-expression analysis between potato and tomato coupled with chemical profiling, Itkin et al. (2013) identified 10 SGA biosynthesis genes, including four genes encoding UDP-dependent glycosyltransferases (*GAME1*, *GAME2*, *GAME17*, and *GAME18*), 5 genes coding for cytochrome P450 monooxygenases (hereafter abbreviated as CYP) (*GAME4*, *GAME6*, *GAME7*, *GAME8*, and *GAME11*), and one aminotransferase gene (*GAME12*). Furthermore, eight of these genes were found to be present as gene clusters on chromosome 7 and 12. These genes were found to be involved in SGA biosynthesis by suppressing or overexpressing them in transgenic plants and chemically profiling them.

## Cytochrome P450 monooxygenases

Cholesterol is thought to be oxygenated at the C-16, C-22, and C-26 positions to form various SGAs. It is known that multiple CYPs are involved in the oxidation reactions in brassinosteroid biosynthesis (Ohnishi et al. 2009), suggesting the involvement of CYPs in oxygenation of cholesterol in SGA biosynthesis. Three candidate *CYP* genes (*PGA1/CYP72A208/GAME7*, *PGA2/CYP72A188/GAME8*, and *PGA3/CYP88B1/GAME4*), which are highly expressed in sprouts with high SGA accumulation, were selected from the potato expression database (Spud DB, http://solanaceae.plantbiology.msu.edu/pgsc_download.shtml), and transgenic potato plants in which their expression was suppressed using RNA interference (RNAi) resulted in a significant reduction of SGA content (Umemoto et al. 2016). Functional analysis revealed that *PGA2* encodes cholesterol 22-hydroxylase and that *PGA1* encodes a 26-hydroxylase that converts 22-hydroxycholesterol to 22,26-dihydroxycholesterol (Figure 2). On the other hand, the catalytic function of PGA3 remains unclear, but the *PGA3*-suppressed plants accumulated a non-nitrogen-containing furostanol-type steroidal saponin (neoindioside D), suggesting that PGA3 is involved in the 26-oxidation to form a 26-aldehyde intermediate (Figure 2).

## 2-Oxoglutarate-dependent dioxygenase, 16DOX

Three CYPs mentioned above have been identified as being involved in SGA biosynthesis, whereas the enzyme responsible for hydroxylating at the C-16 position remained unknown. Therefore, we focused on 2-oxoglutarate-dependent dioxygenases (hereafter abbreviated as DOXs) as another oxygenase candidates and selected the *16DOX* gene (Nakayasu et al. 2017). When the *16DOX* expression was suppressed via RNAi, the SGA content significantly decreased, and the glycoside of 22,26-dihydroxycholesterol accumulated. Functional analysis revealed that the recombinant 16DOX enzyme is a 16α-hydroxylase that specifically introduces a hydroxy group at the C-16α position of 22,26-dihydroxycholesterol (Figure 2).

## Aminotransferase, PGA4

Tracer experiments suggested that a nitrogen atom is added to the C26 position and that an aldehyde intermediate is involved in the nitrogen addition (Ohyama et al. 2013). Therefore, it was expected that the C-26 amination reaction would be catalyzed by an aminotransferase. Based on the identification of the *pAmt* gene which is involved in the biosynthesis of the alkaloid capsaicin in *Capsicum annuum* (Lang et al. 2009), *PGA4* showing a high homology to *pAmt* was selected as a candidate aminotransferase gene (Nakayasu et al. 2021b). The SGA content significantly decreased in *PGA4*-suppressed potato plants via RNAi, and similar to *PGA3*-suppressed plants, furostanol saponin (neoindioside D) accumulated (Figure 2). Enzymatic analysis revealed that PGA4 catalyzes transamination at position C-26 of 22-hydroxy-26-oxocholesterol with γ-aminobutyric acid as an amino donor (Nakayasu et al. 2021b).

## Dioxygenase for potato solanidane synthesis, DPS

SGAs are divided into solanidane and spirosolane based on the structure of their skeleton derived from the side chain of cholesterol (Figure 1). α-Solanine is a representative compound of solanidane, and solanidane production is almost limited to cultivated and wild potatoes. On the other hand, spirosolane is found in a wide range of plants in the Solanaceae family, and α-tomatine in tomatoes and α-solasonine in eggplants are well known spirosolane-type SGAs. The identified biosynthesis genes mentioned above are the genes common to both potatoes and tomatoes, and therefore, to identify the gene that creates this structural difference in the EF-ring structure, a candidate gene should be expressed specifically in potatoes. The *DPS* gene (*Dioxygenase for Potato Solanidane synthesis*), that is strongly expressed in potato sprouts but not expressed in tomatoes, were selected as a candidate (Akiyama et al. 2021b). Suppression of the *DPS* expression resulted in decreasing the amount of α-solanine and α-chaconine and accumulating the spirosolane-type SGA, α-solamargine. Biochemical analysis revealed that DPS catalyzes the ring-rearrangement from spirosolane to a solanidane, zwittersolanine, via C-16 hydroxylation (Figure 2). From these results, DPS contributes to the emergence of toxic solanidane glycoalkaloids in potato and the chemical diversity in Solanaceae.

## Modification of AB-rings

The structural difference between α-tomatine and dehydrotomatine, major SGAs in tomatoes, is the presence or absence of double bonds between C-5 and C-6, and the four reaction steps including C-3 oxidation, isomerization, C-5α reduction, and C-3 reduction are involved in the conversion between them. Two genes, *Sl3*β*HSD1* and *SlS5*α*R2*, responsible for these four reactions have been identified (Akiyama et al. 2019; Lee et al. 2019) (Figure 2). Sl3βHSD1 is a multifunctional enzyme that possesses the activities of 3β-hydroxysteroid dehydrogenase/Δ^5,4^ isomerase when acting on dehydrotomatidine to form tomatid-4-en-3-one and also shows the activity of 3-ketosteroid reductase when acting on tomatid-3-one to produce tomatidine (Lee et al. 2019). Sonawane et al. (2018) also reported that *GAME25* encodes 3β-hydroxysteroid dehydrogenase/Δ^5,4^ isomerase, which acts not only on diverse steroidal alkaloid aglycone substrates but also on steroidal saponin aglycones.

The 5α-reduction step is thought to be catalyzed by a steroid 5α-reductase, and Arabidopsis *DET2* catalyzes the NADPH-dependent reduction of the Δ^4,5^ double bond in brassinosteroid biosynthesis (Noguchi et al. 1999). In tomato, there are two *DET2* homologs, *SlS5*α*R1* and *SlS5*α*R2*, and CRISPR/Cas9-mediated knockout of either *SlS5*α*R2* or *SlS5*α*R1* revealed that disruption of *SlS5*α*R1* did not affect the endogenous SGA levels and that *SlS5*α*R2*-knockout tomato hairy roots showed drastic reduction in the α-tomatine level and significant accumulation of dehydrotomatine (Akiyama et al. 2019). These results indicate that *SlS5*α*R2* is responsible for the 5α-reduction step in α-tomatine biosynthesis (Figure 2) and that *SlS5*α*R1*, which encodes a functional steroid 5α-reductase, does function in general sterol biosynthesis but not significantly contribute to α-tomatine biosynthesis.

## Dioxygenases in α-tomatine metabolism

**Figure 3:**
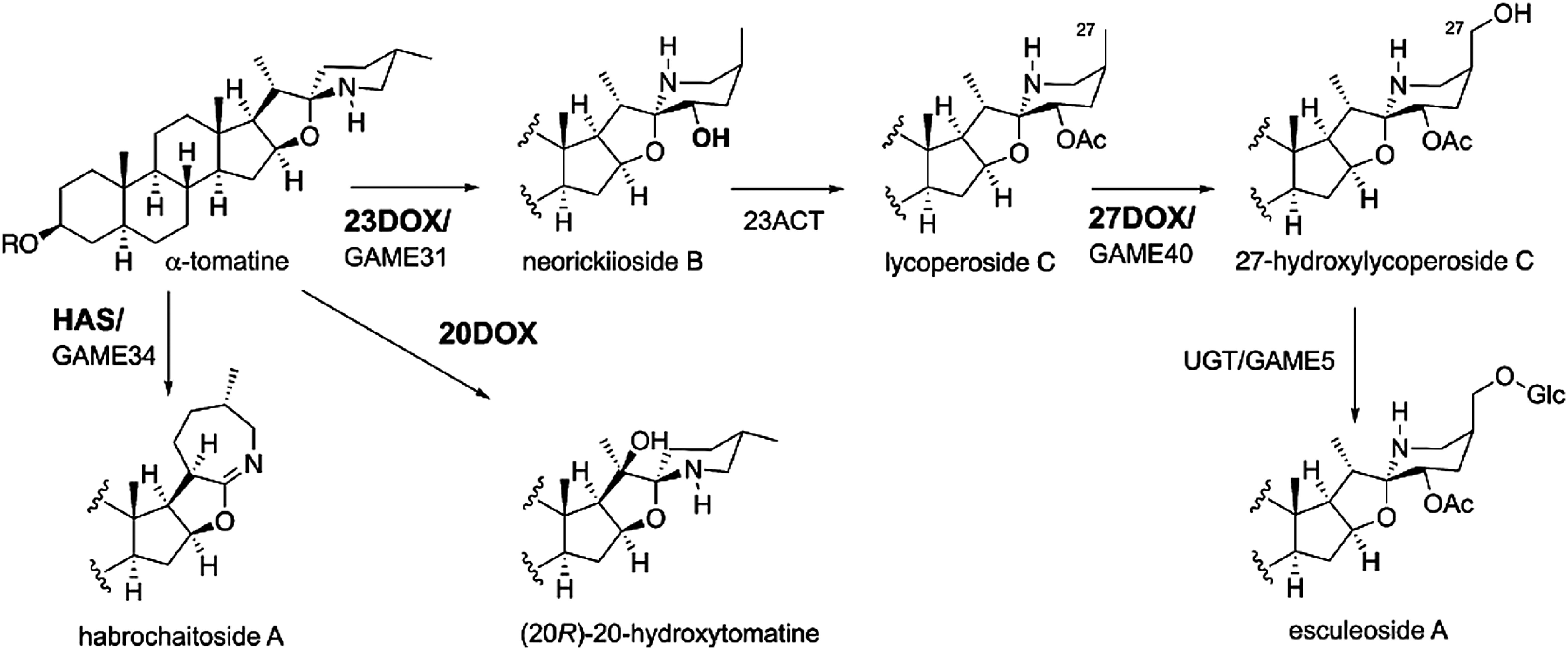
Figure 3. α-tomatine metabolism by 2-oxoglutarate-dependent dioxygenases in cultivated and wild tomatoes.

α-Tomatine is a toxic and bitter compound in tomatoes, but ingestion of tomato fruits rarely results in food poisoning. This is because detoxification metabolism of α-tomatine occurs in tomato fruits. The content of α-tomatine in the fruits decreases during ripening and is not accumulated in the fully ripe, red stage. In contrast, esculeoside A, a tasteless and nontoxic SGA, increases during ripening. It is known that α-tomatine is converted to esculeoside A through 23-hydroxylation, 22-isomerization, 27-hydroxylation, acetylation of 23-hydroxy group, and glycosylation of 27-hydroxy group during ripening, and three metabolic intermediate structures have been determined (Iijima et al. 2008). The candidate gene for the initial hydroxylation reaction at C-23, which is responsible for the detoxification metabolism, was selected as *Sl23DOX*, a *DOX* family gene whose expression level increases during ripening (Nakayasu et al. 2020). The recombinant Sl23DOX enzyme was shown to catalyze C-23 hydroxylation of α-tomatine, followed by 22-isomerization, to generate neolycopersenoside B (Figure 3). Cárdenas et al. (2019) also reported similar results for the same gene, named *GAME31*. The conversion to tasteless and nontoxic esculeoside A during ripening are important events for us to safely and deliciously consume tomato fruits, and therefore it is likely that *Sl23DOX* expression during fruit ripening was positively selected during tomato domestication.

The *E8* gene is well known as an inducible *DOX* gene in response to ethylene during fruit ripening (Lincoln et al. 1987), and recently, E8 was found to function as a C-27 hydroxylase of lycoperoside C to produce 27-hydroxylycoperoside C in the metabolic detoxification of α-tomatine, and is designated as Sl27DOX (Akiyama et al. 2021a) (Figure 3). Sonawane et al. (2022) also reported similar results for the same gene, named *GAME40*.

## Diverse SGAs by duplicated dioxygenases

It was found that tandemly duplicated *DOX* genes with high similarity to *Sl23DOX* or *Sl27DOX* are present on tomato chromosome 1 or chromosome 9, respectively (Akiyama et al. 2021a, 2022). Functional analysis of two Sl23DOX homologs on tomato chromosome 1 showed that they accepted α-tomatine as a substrate as does Sl23DOX, but gave the reaction products different from 23-hydroxytomatine. Structural analysis of the reaction products revealed that they are 20-hydroxytomatine and habrochaitoside A, and these Sl23DOX homologs are found to encode α-tomatine 20-hydroxylase (20DOX) and habrochaitoside A synthase (HAS), respectively (Figure 3) (Akiyama et al. 2022). Sonawane et al. (2022) also reported similar results for HAS, named *GAME33*. *20DOX* and *HAS* are rarely expressed in cultivated tomatoes, but are expressed in fruits and roots of wild tomato *Solanum habrochaites* accession LA1777, resulting in accumulation of 20-hydroxytomatine and habrochaitoside A in these tissues. Thus, functional divergence of α-tomatine-metabolizing DOX enzymes is attributed to tandem gene duplication and the neofunctionalization of catalytic activity and gene expression, which may contribute to a driving force of SGA structural diversity in the tomato clade.

## Genome editing

The majority of currently cultivated potato varieties are tetraploid and possess four alleles of the targeted SGA genes on the genome. Therefore, it is necessary to destroy the genes in all four alleles to block SGA biosynthesis. In crops with seed propagation, such as rice and tomato, it is possible to introduce a mutation into one allele at random on the genome using mutagens such as EMS and accumulate mutations through backcrossing. On the other hand, because potato is propagated asexually, crossbreeding results in offspring with varied traits, making it difficult to inherit the superior traits of the parent variety. Additionally, it takes a long time to accumulate a mutation from one allele to four alleles. Therefore, it was difficult to create a potato that does not produce SGA. However, with the development of a new breeding technology called genome editing, it has become possible to create a potato that does not produce SGA. Artificial restriction enzymes can be freely designed to cut the target sequence, enabling the destruction of the target gene. Here, we introduce genome editing of the SGA biosynthesis gene can be examined using the most widely used artificial restriction enzymes in genome editing, TALEN and CRISPR/Cas9 (Figure 2). In an example using TALEN, SGA-reduced potato was created in the current generation in which all alleles of the *SSR2* gene in the tetraploid potato were disrupted as a result of genome editing targeting the *SSR2* gene (Sawai et al 2014; Yasumoto et al. 2020, 2019). Genome editing of *16DOX* using CRISPR/Cas9 was examined in the hairy root transformation system, and no SGA was detected in the *16DOX* knockout hairy roots (Nakayasu et al. 2018a). As described above, it is known that suppression of the expression of *PGA3* or *PGA4* resulted in accumulation of steroidal saponins instead of toxic SGAs, and therefore, genome editing of them can lead to accumulation of pharmaceutically useful saponins in potato and tomato.

## Conclusion

Potatoes and tomatoes accumulate a large amount of SGA, which can exceed 1% of the dry weight depending on the plant part. SGA exhibits toxicity to a wide range of organisms, and it is thought to be involved in disease and pest resistance. In mutant strains of tomatoes in which the transcription factor JRE4, which comprehensively controls SGA biosynthesis genes, is disrupted, SGA content decreases, and susceptibility to herbivorous insects increases (Nakayasu et al. 2018b). Recent studies have also shown that α-tomatine secreted from tomato roots can change the rhizosphere bacterial community and increase microbial strains that potentially exhibit disease suppression and growth promotion effects in plants (Nakayasu et al. 2021b). SGAs are widely observed compounds in the Solanaceae family, not limited to potatoes and tomatoes, and its structure is highly diverse. Through studying on the biosynthesis mechanism of SGAs as described in this review, it will be possible to elucidate why *Solanum* plants produce diverse SGAs.

## Abbreviations

CYP: cytochrome P450 monooxygenase
DOX: 2-oxoglutarate-dependent dioxygenase
SGA: steroidal glycoalkaloid
